# Niche differentiation in rainforest ant communities across three continents

**DOI:** 10.1002/ece3.5394

**Published:** 2019-07-17

**Authors:** Michael E. Grevé, Mickal Houadria, Alan N. Andersen, Florian Menzel

**Affiliations:** ^1^ Institute of Organismic and Molecular Evolution (IOME), Faculty of Biology University of Mainz Mainz Germany; ^2^ Animal Population Ecology, Animal Ecology I, Bayreuth Center of Ecology and Environmental Research (BayCEER) University of Bayreuth Bayreuth Germany; ^3^ Biology Centre of Academy of Sciences and Faculty of Science, Institute of Entomology University of South Bohemia Ceske Budejovice Czech Republic; ^4^ Tropical Ecosystems Research Centre CSIRO Ecosystem Sciences Darwin Northern Territory Australia; ^5^ Research Institute for the Environment and Livelihoods Charles Darwin University Darwin Northern Territory Australia

**Keywords:** coexistence mechanisms, community structure, Formicidae, interspecific competition, niche partitioning

## Abstract

A central prediction of niche theory is that biotic communities are structured by niche differentiation arising from competition. To date, there have been numerous studies of niche differentiation in local ant communities, but little attention has been given to the macroecology of niche differentiation, including the extent to which particular biomes show distinctive patterns of niche structure across their global ranges. We investigated patterns of niche differentiation and competition in ant communities in tropical rainforests, using different baits reflecting the natural food spectrum. We examined the extent of temporal and dietary niche differentiation and spatial segregation of ant communities at five rainforest sites in the neotropics, paleotropics, and tropical Australia. Despite high niche overlap, we found significant dietary and temporal niche differentiation in every site. However, there was no spatial segregation among foraging ants at the community level, despite strong competition for preferred food resources. Although sucrose, melezitose, and dead insects attracted most ants, some species preferentially foraged on seeds, living insects, or bird feces. Moreover, most sites harbored more diurnal than nocturnal species. Overall niche differentiation was strongest in the least diverse site, possibly due to its lower number of rare species. Both temporal and dietary differentiation thus had strong effects on the ant assemblages, but their relative importance varied markedly among sites. Our analyses show that patterns of niche differentiation in ant communities are highly idiosyncratic even within a biome, such that a mechanistic understanding of the drivers of niche structure in ant communities remains elusive.

## 1 INTRODUCTION

The principle of limiting similarity is one of the central assumptions of niche‐based community ecology, stressing the importance of niche differentiation as the central mechanism of species coexistence (Chase & Leibold, 2003; Hutchinson, 1959). According to niche theory, species with identical niches cannot coexist in a stable equilibrium due to competitive exclusion (Lovette & Hochachka, 2006; Macarthur & Levins, 1967; Sanders, Lessard, Fitzpatrick, & Dunn, 2007). Conversely, interspecific competition is reduced if species occupy niches that differ in any dimension, such as time (Albrecht & Gotelli, 2001; Houadria, Salas‐Lopez, Orivel, Blüthgen, & Menzel, 2015; Santini, Tucci, Ottenetti, & Frizzi, 2007), space (Tanaka, Yamane, & Itioka, 2010), or diet (Feldhaar, Gebauer, & Blüthgen, 2010; McKane et al., 2002). Niche differentiation also reduces competition between species in nonequilibrial communities (Kingston, Jones, Zubaid, & Kunz, 2000; Leibold & McPeek, 2006) and can evolve in response to intraspecific competition (Bolnick, 2001; Maret & Collins, 1997). However, some studies also reported increased niche breadth in response to competition (Bolnick et al., 2010).

Due to intense competition between species (Hölldobler & Wilson, 1990), ants are an ideal taxon to study how species partition their realized niches in the presence of competitors. Many behaviorally dominant ant species displace others from high‐quality resources and even from their entire territories (Blüthgen & Fiedler, 2004a; Hölldobler, 1983; Parr & Gibb, 2010). Being highly diverse, and present in nearly all terrestrial ecosystems, ants encompass a major proportion of terrestrial faunal biomass and play key roles in many ecosystem processes (Folgarait, 1998). Local ant species richness can be extremely high, especially in tropical lowland forests, where several hundred species can occur within a few hectares (Floren & Linsenmair, 2005; Mezger & Pfeiffer, 2011). Many of the functional roles played by ants relate to food consumption (Houadria et al., 2016), which influences rates of nutrient cycling, the dynamics of prey populations, defense of plants against herbivores, and seed dispersal services (Ness, Moon, Lach, & Abbot, 2010; Philpott, Perfecto, Armbrecht, & Parr, 2010).

Ants often show niche differentiation that separates foragers of different species in time (Devoto, Bailey, & Memmott, 2011; Harvey, Dorman, Fitzpatrick, Newman, & McLean, 2012; Lynch, Balinsky, & Vail, 1980; Stuble et al., 2013) or space (Baccaro, De Souza, Franklin, Lemes landeiro, & Magnusson, 2012; Brühl, Gunsalam, & Linsenmair, 1998; Philpott & Armbrecht, 2006). Ants often also show substantial dietary niche differentiation (Menzel, Staab, Chung, Gebauer, & Blüthgen, 2012; Santamaria, Armbrecht, & Lachaud, 2009). Most ant species are generalist scavengers and predators. Some heavily relyon carbohydrate‐rich liquids provided by plants or sap‐feeding trophobionts (Davidson, Cook, & Snelling, 2004). However, many ant species are specialized on a specific resource like termites (Mill, 1984), seeds (Carroll & Janzen, 1973), or fungi (Quinlan & Cherrett, 1979). In habitats where nitrogen is limited, some species even feed on bird feces (Blüthgen & Feldhaar, 2010). Dietary differentiation between species is at least partly due to specialized foraging behavior rather than differential nutritional needs. For example, living insects contain largely similar nutrients to dead ones, but morphological and behavioral specialization on them can reduce competition with other species.

Despite the ubiquity of niche differentiation in ant communities, and the many studies addressing multiple niche dimensions (e.g., Chew, 1977; Davidson, 1977; Bernstein, 1979; Lynch et al., 1980; Torres, 1984; Kaspari & Weiser, 2000; Knaden & Wehner, 2005; Andersen, Arnan, & Sparks, 2013), the relative importance of the different niche dimensions remains largely unknown. Moreover, the relative importance of niche differentiation as a driver of species richness has been questioned (Andersen, 2008), especially in highly diverse communities, where niche differentiation does not appear sufficient to explain the coexistence of all species (Andersen et al., 2013; Houadria et al., 2015; Stuble et al., 2013). Little attention has been given to the macroecology of niche differentiation, addressing the extent to which the relative importance of different niches dimensions can be predicted by climate and habitat structure. It is unknown, for example, if ant communities within any particular habitat type show similar niche structure across different biogeographic regions, due to similar patterns of resource availability.

Here, we analyze the niche structure of tropical rainforest ant communities across five sites on three continents, focussing on the two key niche dimensions of diet and foraging time. Using a standardized sampling design with high spatial replication, we document the degree of dietary and temporal specialization of each species. Our aims were, firstly, to elucidate the relative importance of dietary and temporal niche differentiation for ant species composition. To this end, we conducted comprehensive analyses of overall dietary and temporal niche structure within communities. In addition, we studied dietary and temporal specialization for each species separately to test whether sites differ in number or proportion of specialized species. Our second aim was to use species co‐occurrence in pitfalls and at baits to detect patterns of competition for food. These results were compared between sites on different continents, including primary and secondary forests, to determine whether the observed patterns are consistent across different biogeographic regions with independently evolved ant communities and subject to different levels of disturbance.

## 2 MATERIAL AND METHODS

### 2.1 Study sites

We sampled five rainforest sites on three continents, comprising:

- Two Neotropical forests in French Guiana—a primary forest in Les Nouragues Natural Reserve (Neotropical Primary Forest—NPF) and a secondary forest fragment in Campus Agronomique, Kourou (Neotropical Secondary Forest—NSF)
- Two Paleotropical forests in Sabah, Malaysian Borneo—a primary forest in the Danum Valley Conservation Area (Paleotropical Primary Forest—PPF) and a secondary forest in the Malua forest reserve (Paleotropical Secondary Forest—PSF)
- An Australian monsoonal forest (AMF) (Holmes Jungle nature reserve, Darwin, secondary rainforest fragment)

Further site information is provided in Appendix S1.

### 2.2 Sampling

The study was based on sampling ants recruiting to seven food resources during the day and night, along with catches in pitfall traps. The sampling was performed with 64 spatial replicates per site. The food resources reflected those naturally available to tropical ants (Houadria et al., 2015): dead, crushed insects (mixture of mealworms and local grasshoppers; scavenging); large prey (living grasshoppers or mealworms; predation); termites (living termites, small prey; predation); sucrose solution (for sugars from floral or extrafloral or fruits); melezitose solution (a common trisaccharide in the honeydew of aphids and other ant‐tended trophobionts; Völkl, Woodring, and Fischer (1999) (both sugar solutions were 20 vol. %; 3 ml soaked on paper tissue); bird feces (chicken feces in all sites except for AMF, where local bird feces were used; coprophagy); seeds (mixture of ground corn and sunflower, barley, soya, millet, lin, dari, *Phalaris,* and grass seeds; granivory). Being holometabolous, ants need amino acids or proteins largely for larval growth, while the adult metabolism largely requires carbohydrates (Blüthgen & Feldhaar, 2010; Nation, 2002). Thus, our baits reflected resources largely required for raising brood as well as resources mostly important for adult metabolism. Although novelty or rarity can bias the attractiveness of a resource (Kay, 2004), we believe the resources offered were common and known to the ants enough such that these effects should play a minor role.

Baiting was conducted at 64 points arranged in 4 × 4 grids with 10 m spacing, with four such grids at each site. The four grids were separated by 50–300 m. Ten meters is the recommended distance between sampling points for ground‐dwelling ants (Agosti & Alonso, 2000). Furthermore, ant beta diversity (Sorensen index) did not differ between neighboring grid points, non‐neighboring grid points of the same grid, and different grids (data not shown). To reduce habitat variation between grid points, we took care to avoid forest gaps, that is, all points were under a closed canopy and on flat terrain. Each food resource was presented at each point for 90 min, once at night and once during the day. All food resources were presented in circular plastic boxes with paper tissue at the base and slit‐shaped openings (1 cm height and 8 cm length) on opposite sides to allow access to ants. Only one resource was presented at a grid point at any given time to avoid any interference between different baits. After the 90‐min period, all ants occurring at the resource were collected. Pitfall traps were operated for three 10‐hr periods between 20h00 and 6h00 (nocturnal traps) or 7h00 and 17h00 (diurnal traps) over three consecutive days when no food resources were presented, such that we obtained a total of 30h of pitfall sampling per grid point and per time of day. Pitfall data were used to assess temporal niches, co‐occurrence, and background ant diversity. Sampling was conducted between February 2012 and December 2014 (Appendix S1).

### 2.3 Statistical analyses

For statistical analysis, we used two types of data: frequency (total number of occurrences at baits) and incidence (number of grid points out of 64 per site where an ant species was found). Only for the analyses of species‐specific temporal specialization, we used frequencies based on pooled data for baits and pitfalls.

### 2.4 The relative extent of temporal and dietary niche differentiation

We quantified daily time of activity and diet as two factors structuring ant communities. Spatial effects, that is, turnover between grid points, will also influence species richness and composition. We analyzed which of these factors had the strongest effect on the community structure and compared the effect sizes between the sites.

To this end, we performed a PERMANOVA which allows to simultaneously assess the importance of diet, time, and spatial variation. Furthermore, it allows to test whether there are interactions between the two niche dimensions—for example, whether dietary differentiation differs between day and night. Since each bait was presented at each time of day at each of the 64 grid points, we could account for potential spatial heterogeneity using this approach by incorporating grid point identity in the analysis. At the same time, we could use grid point information to estimate the effect of spatial heterogeneity compared to effects of different food sources or times of day. Due to the standardized experimental setup, we could exclude that any differences between sites or niche dimensions were due to differences in statistical power. We analyzed niche differentiation, separately for each site with a PERMANOVA (software PRIMER 6.1.14 and PERMANOVA+, Primer‐E Ltd.) for which we used frequency data for each ant species, separately for all food resources, grid points, and times of day. The PERMANOVA with 999 permutations had the fixed factors “food source type” and “time of day” and the random factor “grid point.” The percentage of explained variance (sum of the squared deviation per factor divided by the total sum of squares) was used to compare effect sizes between the two niche dimensions and between the sites. The strength of this approach is that the relative roles of diet, time, spatial variation, and their interactions can be easily compared within a single comprehensive analysis. Community composition was visualized using nonmetric multidimensional scaling (NMDS using Bray–Curtis dissimilarity), based on species frequency data.

### 2.5 Dietary and temporal niche overlap

We analyzed whether species were more similar in their preferences than would be expected from random by analyzing niche overlap (as suggested in Fowler, Lessard, & Sanders, 2014) using null model analyses (EcoSim version 7.0, Gotelli and Entsminger (2004), Fowler et al. (2014)). We created two matrices per site in which each row represented a different species and each column represented a different food resource × time combination. The matrices contained the number of times each species was found on the given food resources or time of day. We analyzed niche overlap using Pianka's index (Pianka, 1973), which quantifies niche overlap ranging from 0 (indicating no overlap) to 1 (complete overlap) for each species pair. We simulated 1,000 matrices using RA3. This randomization algorithm retains niche breadth but randomizes which particular resource states are utilized. We chose this algorithm since we offered all food resources at day and night and thus, at all grid points, there was equal access to all resources. Using this model, we tested whether the observed mean niche overlap significantly differed from random.

To compare the effect sizes of niche overlap and co‐occurrences (see 2.8) between sites, we used the simulations to compute the standardized effect size (SES) of niche overlap and co‐occurrences as SES = (*I*_obs_–*I*_sim_)/*s*_sim_ with *I*_obs_ as the observed index (niche overlap or C‐score), *I*_sim_ as the mean simulated index, and s_sim_ as the standard deviation of the simulations, following Gotelli and Ellison (2002). SES values larger than 1.96 or lower than –1.96 indicate significant effects. To compare the effect sizes between the sites, we plotted the SES of niche overlap and co‐occurrences, irrespective of whether SES values were significant or not.

### 2.6 Species‐specific food specialization

We analyzed dietary and temporal specialization for each species separately and calculated absolute and relative preferences using a “hotlink” analysis (see below). The relative extent of temporal and dietary niche differentiation was compared between sites based on effect sizes and numbers of specialized species.

Food specialization was calculated for each species with an incidence ≥ 5 (i.e., number of different grid points where the species was found; total number of species = 109; ranging from 11 – 31 per site). For each species *n*, its food specialization index (*fs*_n_) was calculated as $fs_{n}=\sum p_{i,n}^{2}$, with *p*_i,n_ being its frequency on food resource *i* divided by its total frequency (analogous to the Simpson index). We calculated *fs*_n_ 1,000 times based on 5 randomly drawn occurrences, to avoid a bias caused by differences in overall frequency of common and rare species. *fs*_n_ ranges from 0 (for a generalist) to 1 (for a dietary specialist). We compared *fs*_n_ values across sites (as independent variable) using a linear model (LM), assuming normal distribution.

While *fs*_n_ describes the degree of food specialization of a species, it does not provide information about the type of food resource that a species prefers. This was evaluated by calculating absolute and relative food preferences of each species with a total incidence ≥ 5. The “absolute preference” of a species indicates whether a certain resource is more attractive to this species than other resources. In contrast, its “relative preference” indicates whether a certain resource is more attractive to this species compared to the other species. The latter is especially relevant given that many ant species were attracted to the same resources.

For the *absolute* food preferences, for each species we calculated a null model based on the incidence per food resource (pooled for day and night). In 1,000 permutations, we randomly assigned all occurrences to the seven food resources and compared it with the real incidences per food resource. If the species occurred more often on a food source than expected by random (α = 0.025), the resource was defined as significantly preferred.

*Relative* food preferences were calculated based on the “hot link” analyses from Junker, Höcherl, and Blüthgen (2010). In contrast to the absolute preferences, we constructed a bipartite network with species incidences versus the seven resources. The “hot link” analysis compared the number of occurrences of a species on a resource relative to the occurrences of the whole community on this resource. It revealed whether a species occurred more often on a resource than other species, even if it was an unattractive resource seldom visited by most species. Thus, relative preferences give a clearer picture about (realized) niche differentiation that is unbiased by overall resource attractiveness. Here, a null model was created which randomly shuffled species occurrences among the resources, but with total species‐wise incidences kept constant and equal to the real data (Junker et al., 2010). Based on 1,000 randomizations, the realized number of occurrences of a species on each food source was compared with the whole number of occurrences of all species on each food source. If a species were more common on a food source than expected, it was defined as a relative preference (α = 0.025). Note that all the preferences reflect “realized” rather than “fundamental” preferences since they are based on data in the presence of competitors. We use the term “preference” to distinguish these data (on the identity of a preferred resource type) from “specialization,” which is a single value ranging from generalization to specialization.

We compared the numbers of species with and without absolute or relative food preferences across sites using chi‐squared tests. Since less common species are predicted to have a low impact on their community and for a higher clarity of the results, we show only the analysis for the most common species that together comprised 80% of all occurrences (see Table S1 for an analysis of species with incidence ≥ 5). As a site‐level measure of overall niche differentiation, we divided the total number of significant absolute or relative preferences by the number of species.

### 2.7 Species‐specific temporal specialization

For each species *n* with a frequency ≥ 5 (*N* = 155), we calculated its temporal niche *tn*_n_ as $tn_{n}=2*\frac{{\text{freq}}_{{\text{day}}_{n}}}{{\text{freq}}_{{\text{day}}_{n}}+{\text{freq}}_{{\text{night}}_{n}}}-1$, with ${\text{freq}}_{{\text{day}}_{n}}$ as the total number of occurrences of species during day and ${\text{freq}}_{{\text{night}}_{n}}$ during the night (Houadria et al., 2015) on food resources and in pitfall traps. *tn*_n_ ranges from −1 for purely nocturnal to + 1 for purely diurnal species. A species was considered specialized if its day and night frequency significantly differed from random according to a chi‐squared test. We compared the temporal niches (*tn*) across the sites with a LM (with *tn* as dependent and site as independent variable). In contrast to the temporal niche, temporal specialization* ts* was calculated as its absolute value ($ts_{n}=\left|tn_{n}\right|$), being 0 for unspecialized and 1 for maximally specialized species. We compared species‐specific temporal specialization across sites using two approaches. Firstly, *ts* was calculated for the same set of species and compared across sites using a LM (with *ts* as dependent and site as independent variable). Secondly, we compared the proportion of temporally specialized species per site with a chi‐squared test.

Finally, we determined whether a species was relatively more frequent during day or night compared to the whole community, by conducting the “hot link” analysis for temporal niche differentiation. This was necessary to reveal deviations from the community average, since, for example, more ant species tend to be active during the day than at night (Houadria et al., 2016).

### 2.8 Overall co‐occurrences and co‐occurrences per resource type and time of day

We performed co‐occurrence analyses to find patterns of spatial segregation (pitfalls) and resource monopolization (food resources) within a community. The standardized effect sizes for niche overlap in diet and time were then compared to overall spatial co‐occurrence. Co‐occurrence was assessed based on two datasets, each time using a species x grid point matrix with presence/absence data (day and night pooled) for all species. Firstly, we calculated co‐occurrences based on pitfall data, that is, unaffected by competition for food resources. Secondly, we calculated co‐occurrence based on baiting data only, separately for each food resource type and time of day, that is, conducted 14 analyses per site (total *n* = 70). This approach allowed a comparison of spatial segregation at food sources (i.e., bait monopolization) between resource types and times of day. Note that the goal of these bait‐based analyses was not to quantify whether species would co‐occur in the same territory or foraging range, but rather to assess whether ants would tend to monopolize baits and displace others from the same bait. Ants frequently compete for highly attractive resources but may show less competitive displacement on less attractive resources (Blüthgen & Fiedler, 2004a). Hence, by estimating monopolization rates for each food resource (via co‐occurrence analysis), we could estimate the degree of competition for different resources. We obtained standardized effect sizes (SES) that were then compared between sites and food source using a LM.

For all these analyses, co‐occurrence was quantified using the C‐score as implemented in EcoSim. We simulated 5,000 random communities, where the occurrences of each species were randomly assigned to the grid points, such that the total number of occurrences per species equaled those in the original matrix. Each grid point had the same probability of being assigned an ant occurrence (fixed‐equiprobable algorithm, Gotelli & Ellison, 2002). This algorithm was chosen since all grid points were in a rather homogeneous habitat without clearly noticeable differences in habitat structure. Furthermore, all baits were presented at all grid points, such that any spatial heterogeneity would equally affect all resource types and both times of day. Hence, any heterogeneity in species numbers at baits could have been biologically meaningful; using the fixed–fixed algorithm would therefore rather correct for, and thus conceal potentially important biological patterns.

All LMs and the hotlink analysis were conducted in R version 3.1.2 (R Development Core Team, 2016). LMs were tested using ANOVA (command *Anova,* package *car*).

## 3 RESULTS

### 3.1 Overview

The five ant communities differed strongly in sampled species richness, with totals summed for food resources and pitfalls ranging from 27 (AMF) to 107 (NPF). Species richness varied markedly among the different food resources, from 10–26 species per site for sucrose to 8–16 species for bird feces (Table 1). Numerical dominance varied between sites (see incidence and frequency data provided in Table 3): The most common species in the Australian monsoonal forest AMF (*Pheidole* sp.A) occurred on all grid points, at the paleotropical secondary forest PSF *Lophomyrmex bedoti* occurred on 97% of all grid points, whereas in the neotropical secondary forest NSF, the most common species (*Pheidole subarmata*) occurred on only 67% of all grid points.

**Table 1:** Overview of variation in ant species richness among sites, sampling methods, resources, and time of day. The table gives the total species richness (baits and pitfalls pooled), species richness on food resources and in pitfalls. Furthermore, the table shows the number of species with incidence ≥ 5 and, in brackets, the total number of species per food source (food resources only), as well as the number of species with frequency ≥ 5 and, in brackets, total number of species, per time of day (food resources and pitfalls)

Site	Total	Food resources	Pitfalls	Sucrose		Melezitose		Crushed insects		Termites		Seeds		Large prey		Bird feces		Day		Night	
AMF	27	16	21	10	(11)	10	(11)	12	(14)	9	(9)	9	(11)	7	(7)	8	(8)	12	(19)	11	(19)
PPF	92	56	81	26	(33)	20	(26)	22	(29)	23	(28)	19	(24)	16	(21)	19	(26)	41	(82)	41	(76)
PSF	85	61	54	15	(29)	15	(28)	14	(24)	10	(19)	10	(20)	14	(15)	11	(20)	21	(61)	23	(60)
NPF	108	50	90	26	(34)	24	(34)	23	(32)	24	(30)	18	(19)	19	(23)	13	(16)	48	(83)	46	(78)
NSF	50	34	47	19	(21)	19	(20)	21	(25)	18	(19)	20	(21)	17	(19)	16	(17)	28	(47)	28	(34)

### 3.2 Effects of diet, time, and space on ant communities

**Figure 1:**
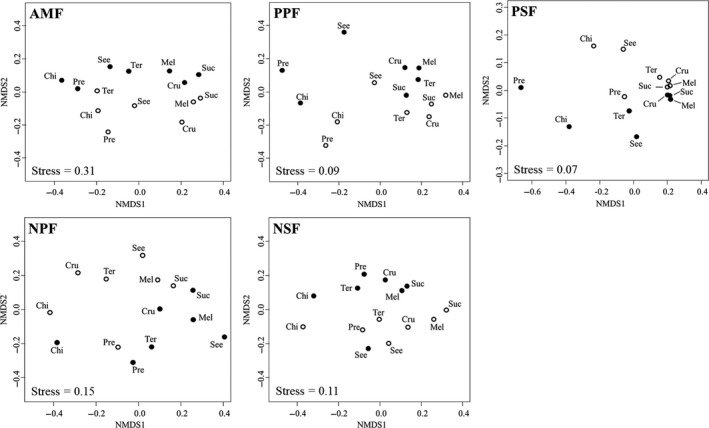
NMDS ordinations (based on presence/absence data; Bray–Curtis dissimilarity) of the ant assemblages attracted to the seven food resources and the two time periods for each of the five sites. Cru—crushed insects; See—seeds; Suc—sucrose; Mel—melezitose; Pre—large prey (live grasshoppers/mealworms); Ter—live termites; Chi—bird feces. Full circles represent nocturnal and empty circles diurnal communities. In addition, the stress level for each NMDS ordination is stated

Ant assemblages were strongly affected by both food resource and time in all sites. Food resource (mean of 39.4%) and time (37.6%) both explained significant amounts of variation overall in ant species composition, but their relative importance varied markedly among sites (Table 2). For example, in PSF food resource explained 66% of variation (pseudo‐*F*_6,378_ = 22.26) and time only 11% (pseudo‐*F*_1,378_ = 3.05), whereas in NSF time explained 55% (pseudo‐*F*_1_ = 25.63) and food resource only 22% (pseudo‐*F*_1,378_ = 9.04). Variation among sites in the relative importance of food resource and time as niche dimensions is illustrated by variation in ant species composition for each food resource x time combination (Fig. 1). For example, in PSF ant assemblages on melezitose, sucrose and crushed insects were highly similar to each other (both for day and night) and cluster together closer than in the other sites. In both neotropical sites, time explained more variance than diet, while in the paleotropical sites (especially PSF), diet had a stronger influence. Notably, the highest percentage of explained variance by diet plus time (including the interaction) was in the less species‐rich AMF. Spatial variation in ant assemblage composition accounted for only 5%–14% of the total variation (Table 2).

**Table 2:** Factors explaining community composition in the five sites. The table shows results of a PERMANOVA that was based on the incidence of each species at each of the seven food sources. It included the fixed factors “food resource” and “time of day” their interaction, and “grid point” as a random factor. The pseudo‐*F* values indicate the effect size of each factor on the ant communities

Site	Food resource (*df* = 6, 378)		Explained variance	Time (*df* = 1, 378)		Explained variance	Grid point (*df* = 63, 378)		Explained variance	Diet: Time interaction (*df* = 63, 378)		Total variance explained by diet, time and their interaction
	Pseudo‐*F*	*p*		Pseudo‐*F*	*p*		Pseudo‐*F*	*p*		Pseudo‐*F*	*p*	
AMF	23.86	**0.001**	45%	15.89	**0.001**	41%	3.2	**0.001**	5%	2.33	**0.004**	89%
PPF	7.86	**0.001**	35%	6.74	**0.001**	33%	3.58	**0.001**	14%	1.19	0.193	73%
PSF	22.26	**0.001**	66%	3.05	**0.009**	11%	3.27	**0.001**	9%	1.75	**0.015**	82%
NPF	8.47	**0.001**	29%	12.72	**0.001**	48%	2.57	**0.001**	7%	2.05	**0.001**	83%
NSF	9.04	**0.001**	22%	25.63	**0.001**	55%	4.95	**0.001**	11%	2.58	**0.001**	83%

Note

Also shown are percentages of variance explained by diet, time, space (i.e., grid point) as well as the total variance explained by the factors: diet + time + the diet:time interaction. Significant *p* values are given in bold.

### 3.3 Niche specialization and overlap

**Figure 2:**
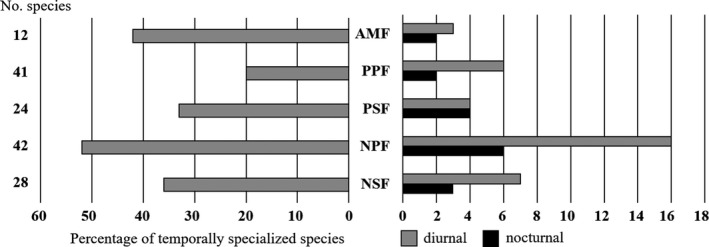
Percentage and number of temporally specialized species on each site (out of a total *N* of 155)

There was no variation among sites in the extent of species‐specific dietary specialization (*fs*) (LM: *F*_4_ = 0.72; *p* = 0.58). The same was true for the proportion of species with absolute food preferences, although this ranged from 19% to 55% (χ^2^ test: χ^2^_4_ = 8.58; *p* = 0.07; Table S3). Similarly, neither temporal specialization (*ts*) (LM: *F*_4_ = 1.92; *p* = 0.11) nor temporal niche (*tn*) (LM: *F*_4_ = 0.81, *p* = 0.52) varied among sites. However, the proportion of absolute temporal specialists differed among sites (χ^2^ test: χ^2^_4_ = 6.39; *p* = 0.011), ranging from 20% in PPF to 44% in NPF (Figure 2). Notably, all sites except PSF harbored more diurnal than nocturnal species (Figure 2). This was true also for relative temporal preferences that accounted for overall community preferences (Table 3).

**Table 3:** Absolute (green) and relative (red) preferences for food and time of day, shown for the most common species (together accounting for 80% of all occurrences on food sources) per site. Incidence (number of grid points) and frequency (on food sources) per species are given additionally. The food resources are sorted by its attractiveness in declining order (see Figure 4a)

Site	Incidence	Frequency	Sucrose		Melezitose		Crushed insects		Termites		Seeds		Large prey		Bird feces		Day		Night	
AMF																				
*Pheidole sp.A*	64	417																		
*Nylanderia *sp.*1*	51	137																		
*Oecophylla smaragdina*	44	118																		
PPF																				
*Lophomyrmex bedoti*	56	252																		
*Carebara *sp.1	50	98																		
*Lophomyrmex longicornis*	27	55																		
*Nylanderia *sp.4	27	41																		
*Tapinoma *sp.1	13	36																		
*Pheidole *sp.6	20	34																		
*Pheidole *sp.5	12	33																		
*Euprenolepis *sp.1	15	25																		
*Recurvidris *sp.2	17	24																		
*Pheidole *sp.40	8	22																		
*Dinomyrmex gigas*	18	21																		
*Carebara *sp.8	15	16																		
PSF																				
*Lophomyrmex bedoti*	62	364																		
*Carebara *sp.1	35	49																		
*Technomyrmex *sp.2	20	34																		
*Myrmicaria *sp.1	10	25																		
*Lophomyrmex longicornis*	9	22																		
*Recurvidris *sp.2	11	18																		
NPF																				
*Pheidole cf. nitella*	52	137																		
*Crematogaster levior*	42	96																		
*Camponotus femoratus*	30	74																		
*Crematogaster limata*	31	61																		
*Pheidole *sp.6	27	42																		
*Solenopsis *sp.15	24	34																		
*Pheidole *sp.8	16	29																		
*Pheidole *sp.28	15	24																		
*Nylanderia *sp.2	16	22																		
*Ectatomma *sp.4	13	21																		
*Pheidole *sp.19	12	20																		
*Solenopsis *sp. 9	13	19																		
NSF																				
*Pheidole subarmata*	43	175																		
*Pheidole pugnax*	40	138																		
*Camponotus *sp.2	51	110																		
*Solenopsis *sp. D2	25	50																		
*Solenopsis *sp. D1	29	43																		
*Nylanderia *sp.1	16	33																		
*Solenopsis *sp.1	18	44																		
*Pheidole zeteki*	23	35																		
*Odontomachus haematodus*	19	30																		
*Pheidole *sp.5	12	31																		
*Crematogaster limata*	13	27																		
*Pheidole *sp.1	13	24																		
Total number of absolute preferences			15		9		9		0		1		1		1		16		3	
Total number of relative preferences			3		4		3		2		6		2		3		11		6	

Note

Based on null model randomizations, a food resource was defined as absolutely preferred (green) if a species foraged on it significantly more often than on other food resources, and as relatively preferred (red) if a species foraged significantly more often on it than the other species within its community. On the right, absolute and relative temporal preferences are shown, based on total frequencies (food sources and pitfalls combined, not shown) per species. At the bottom, the total number of absolute and relative preferences is shown.

**Figure 3:**
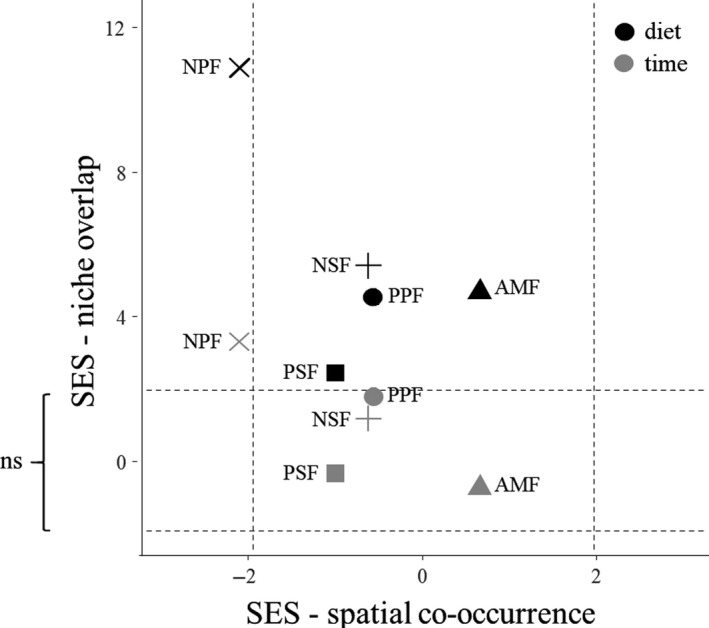
Niche overlap plotted against spatial co‐occurrence. The points represent standard effect sizes (SES) per site for dietary and temporal niche overlap (*y*‐axis) and for spatial co‐occurrence at pitfalls (*x*‐axis). Sites with SES values greater than 1.96 (dashed lines) indicate significant species segregation (*x*‐axis) or higher niche overlap than expected from random (*y*‐axis), respectively. SES values less than −1.96 indicate significant species aggregation (*x*‐axis) or niche partitioning (*y*‐axis)

Dietary niche overlap between species was higher than expected by chance in all five sites (*p*_obs_ > *p*_exp_; *p* < 0.025; Table S2a, Figure 3). In contrast, only the NPF community showed significant temporal niche overlap (Table S2a, Figure 3). Standardized effect sizes for time were significantly smaller than those for diet (paired *t* test: *t*_4_ = 5.03, *p* = 0.0073).

### 3.4 Food preferences

**Figure 4:**
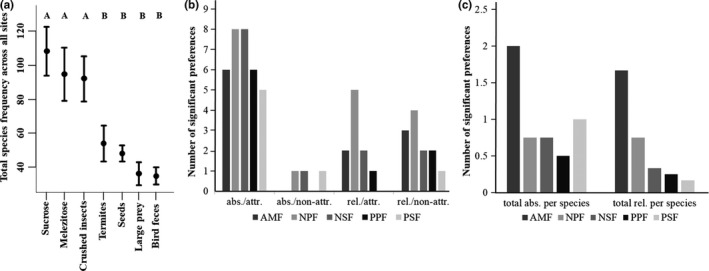
(a) Total frequency per species (mean ± standard error) per resource type at the five sites. Plots with the same letters are not significantly different based on Tukey's HSD. (b) Number of significant absolute (abs.) and relative (rel.) preferences for attractive (attr.) (crushed insects, sucrose, melezitose) and nonattractive (non‐attr.) (bird feces, seeds, living termites, living large prey) food sources per site. A “preference” is defined here as a species that occurred more frequently on a given resource type than expected. Note that a single species can have significant preferences for multiple resources. (c) Number of significant absolute and relative preferences per species and site, summed for all resources

Overall, crushed insects, sucrose, and melezitose were most attractive as measured by their frequencies (Figure 4a). This attractiveness was reflected in the species‐wise preferences: Absolute preferences in any species mostly concerned these three resources (green cells in Table 3; Figure 4b). Few absolute preferences were detected for other resources, with examples including large prey (*Odontomachus haematodus* in NSF), seeds (*Carebara* sp.1 in PSF), or bird feces (*Camponotus femoratus* in NPF, Table 3). However, when we accounted for overall attractiveness by analyzing relative preferences, we detected relative specialization on a broader spectrum of resources. Many species showed relative preferences (red cells in Table 3) for nonattractive resources, which resulted in a more even distribution of preferences across resource types (Table 3, Figure 4b) (Shannon evenness for all absolute preferences across the seven resource types: 0.65; per site: 0.57 ± 0.03; Shannon evenness for all relative preferences: 0.93; per site: 0.61 ± 0.15).

Using this approach, we found strong patterns of dietary niche differentiation among the most common species of each site (Table 3). AMF showed the strongest of niche differentiation, measured by the number of absolute and relative preferences per species (Figure 4c). Here, the most common species (*Pheidole* sp. A) foraged more on seeds and termites compared to the other two most common species, although in absolute terms, it foraged most on crushed insects and sucrose. The second‐most common species, *Nylanderia* sp.1, similarly foraged most on crushed insects, sucrose, and melezitose, but relative to the other two species foraged more on melezitose and sucrose. The third common species, *Oecophylla smaragdina*, fed on large prey more than the other species. Thus, AMF showed a relatively high level of niche partitioning, which we quantified via the number of significant preferences compared to the number of analyzed species. In PPF, PSF, and NPF, dominant species (like *Carebara* sp.1, *Pheidole* cf. *nitella*,* Camponotus  femoratus*) frequently foraged more on less attractive resources like seeds or bird feces. Only in NSF, the three most common species showed no discernible bait differentiation.

### 3.5 Co‐occurrence on food resources and in pitfalls

**Figure 5:**
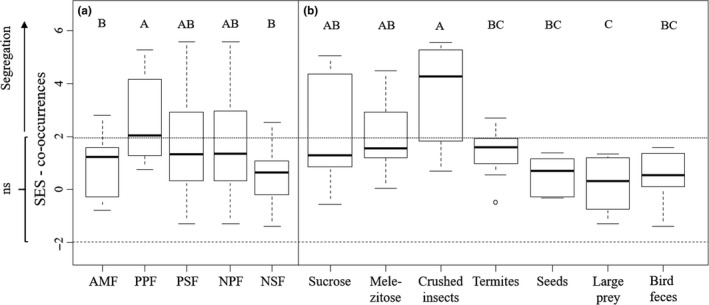
Species co‐occurrence on food resources, shown as standardized effect sizes. Co‐occurrence was calculated separately for each food source and time of day. (a) Co‐occurrence on different sites (*n* = 14 per site [7 baits, 2 times of day]). (b) Co‐occurrence per food source (*n* = 10 per food resource [2 times of day, 5 sites]). Values greater than 1.96 (dashed lines) indicate significant species segregation; values <−1.96 indicate significant species aggregation. Plots with the same letters are not significantly different based on Tukey's HSD comparisons. See Figure 3 for co‐occurrence in pitfalls

We measured spatial segregation on food resources as an indicator for the monopolization of a resource type. There was significant variation among sites in spatial segregation (LM: *F*_4_ = 7.34 *p* < 0.0001; Figure 5a). Paleotropical primary forest (PPF) had the highest level of segregation, which we interpret as strongest degree of competitive exclusion at food sources. The numerically dominant species of PSF and PPF showed not only high frequencies, but also high mean abundances (number of workers) per occurrence and food resource (e.g., *L. bedoti*: 100.6 in PSF, 94.8 in PPF; *Carebara* sp.1:247.36 in PPF), indicating that they were well able to exclude other species from a food resource. In general, over all sites, segregation was highest on the three highly attractive resources (effect of resource type: LM: *F*_6_ = 12.1; *p* < 0.0001; Figure 5b). Time of day did not affect segregation (LM: *F*_1_ = 1.91; *p* = 0.17).

In contrast to segregation at baits, segregation at pitfalls was much lower. Here, standardized effect sizes per site ranged from 0.66 (AMF) to −2.11 (NPF). Thus, co‐occurrence in pitfalls was different from random only at NPF (Table S2b, Figure 3).

## 4 DISCUSSION

In this study, we address the extent to which ant communities in tropical rainforest across different biogeographic regions show consistent patterns of dietary and temporal niche differentiation, and of species co‐occurrence. To our knowledge, this is the first macroecological study of niche differentiation in ant communities, using a consistent sampling methodology to examine the extent to which the relative importance of different niches dimensions is predictable or consistent in species‐rich communities.

### 4.1 The importance of diet and time varies among sites

Both resource type and time of day significantly influenced the composition of ant assemblages at each site. However, their relative importance differed (Table 2). In NSF and NPF, time of day played a larger role than dietary differentiation, while the reverse was true in PSF. Both factors were approximately equally important in the Australian forest (AMF). Thus, the effect of single niche dimensions on community composition seems to be highly idiosyncratic and specific to the site studied.

Variation among sites in the importance of diet and time is reflected by variation in niche preferences of dominant species. For example, the three most abundant species in NPF all showed absolute temporal specialization, whereas none of the three most abundant species in PSF did so (green cells in Table 3). This is consistent with the high impact of time in NPF, but low in PSF. Similarly, the high impact of diet on community structure in PSF and PPF reflects the extremely high abundance of *Lophomyrmex bedoti*, which mostly monopolized attractive resources and thereby caused community differences between attractive and nonattractive resources. Thus, temporal and dietary specialization of dominant species can directly affect overall community patterns, especially given that bait monopolization and competitive exclusion are largely driven by them (Arnan, Gaucherel, & Andersen, 2011; Blüthgen & Fiedler, 2004a; Ellwood, Blüthgen, Fayle, Foster, & Menzel, 2016; Parr & Gibb, 2010). Moreover, their numerical abundance and tendency to monopolize can strongly influence both community structure and the level of spatial segregation. Our findings at PSF and PPF demonstrate that a single dominant species can greatly affect community‐wide patterns of niche partitioning. These effects are idiosyncratic and hard to predict based on community composition alone (Houadria & Menzel, 2017).

Next to diet and time, spatial variation also significantly contributed to community composition, but only accounted for 5%–14% of the variation. Note that, due to the balanced experimental design of our study, the spatial variation could not affect our results concerning relative impacts of the two niche dimensions, niche overlap, preferences, and specialization.

### 4.2 Specialization per species

Despite the different effect sizes for dietary and temporal differentiation, average specialization per species (*fs* and *ts*) did not differ across sites. However, species‐specific values ignore the numerical importance of each species and do not consider specialization relative to the remaining community: Rare species with little effect on community structure had the same weight as common species. Thus, average specialization of a community does not necessarily yield information on the actual importance of a certain niche dimension for community structure. To thoroughly assess the role of a niche dimension, one should take into account each species’ ecological importance and measure “relative specialization,” that is, how different each species is from the remaining community, rather than absolute specialization (Houadria & Menzel, 2017).

### 4.3 High overall niche overlap

Across the entire communities, dietary niche overlap was always higher than expected from random. This is due to three resources (sucrose, melezitose, and crushed insects) that were widely preferred by dominant and rare species. Nevertheless, these seemingly generalistic species showed signs of niche differentiation as revealed by the hotlink analyses: Some species preferentially foraged on otherwise less attractive resources compared to the remaining community. Note, however, that other food specialists might be entirely missing from our study—specialized predators, leaf‐cutters, or fungivores may not have been attracted to the baits at all since we did not offer their main diet. By missing these specialists, we have underestimated overall potential food partitioning.

Besides the dietary niche, one of the sites (NPF) also showed a higher temporal niche overlap than expected. This is probably because, especially in NPF, there are more diurnal than nocturnal species. Compared to NSF, PPF, and PSF, the difference in species richness between day and night was highest for NPF (Table S2 in Houadria et al., 2016).

### 4.4 Niche differentiation despite strong niche overlap

Dietary niche partitioning became more apparent using relative preferences (hot links), which analyze species‐specific preferences relative to the remaining community. They revealed that, while the numerically dominant species also fed on sucrose, melezitose, and crushed insects, some of them foraged significantly more on termites (*Pheidole* sp.A in AMF), seeds (*Pheidole* sp.A in AMF, *Carebara* sp.1 in PPF, *Pheidole* cf. *nitella* and *Pheidole* sp.6 in NPF), and large prey (*Oecophylla* in AMF) compared with the other species. Thus, these species showed significant relative (!) preferences to resources that were less attractive to the other ants. Moreover, certain species were more active at night compared to the remaining community even if they did not show absolute temporal specialization (Table 3). Many previous studies also found niche differentiation in ant communities in dimensions such as seasonal or daily activity pattern (Albrecht & Gotelli, 2001), diet (Blüthgen, Gebauer, & Fiedler, 2003), or daily activity (Santini et al., 2007; Stuble et al., 2013). Thus, niche partitioning can be detected even in rather generalized communities if overall resource preferences are accounted for.

### 4.5 Spatial segregation at baits and pitfalls

Spatial segregation at baits of the same type indicates resource monopolization in this study and, hence, reflects current competition for this resource type. Our data showed strong spatial segregation at attractive baits, but less so at nonattractive baits. This indicates that resource competition depends on the quality of the resource (Blüthgen & Fiedler, 2004b), for example, if extrafloral sugar concentration is lower at night (Anjos et al., 2017). In pitfalls, spatial segregation was not higher than expected, indicating that segregation at baits was not due to spatial heterogeneity or territoriality. The aggregation found in pitfalls of NPF was probably due to the two mutualistic species *Crematogaster levior* and *Camponotus femoratus*, which were among the most common species in this site and always occurred together.

It should be noted that interspecific competition for food may not reflect competition for other resources, such as nest sites (Ellwood et al., 2016; Tanaka et al., 2010). Other mechanisms to reduce competition may be differences in foraging behavior, for example, species particularly good in discovering food sources versus in defending or monopolizing them. Such trade‐offs, however, are likely to differ between sites and may not be present in many habitats (Parr & Gibb, 2012). Due to the high number of baits (total *n* = 4,480), we could not perform behavioral observations or time series (to observe species turnover) for each bait.

Several studies have recently tested for clustering versus overdispersion in relation to phylogenetic relatedness (Blaimer, Brady, Schultz, & Fisher, 2015; Donoso, 2014) or morphological traits (Fichaux et al., 2019) in species‐rich tropical ant communities. All three studies found evidence of phylogenetic or trait clustering rather than limiting similarity; that is, locally co‐occurring species were morphologically more similar or more closely related than expected, suggesting that interspecific competition is rather weak. However, this does not necessarily contradict our evidence of niche differentiation. Ecological niches may not be phylogenetically conserved; rather they may evolve quickly and are likely to be plastic to some degree. For example, in our dataset, *Pheidole* species in NPF and NSF showed different dietary and temporal niches (Table 3)—hence, despite being congeners, they had strongly different niches. Thus, phylogenetic clustering may well coincide with niche differentiation between co‐occurring species.

### 4.6 Highest niche differentiation in the least diverse site

The Australian forest (AMF) was the species‐poorest site and at the same time showed the strongest niche differentiation, both measured as percent explained variance and as the number of significant preferences per species (Figure 4c). Two nonexclusive explanations for this coincidence are plausible: Either the strong patterns are a result of the lower number of rare species compared to the other sites, which are less specialized and dilute overall patterns, or differentiation is really stronger in species‐poor communities. Firstly, niche differentiation is harder to detect for rare species—their lower abundances lower the statistical power. Thus, higher niche differentiation in a species‐poor community may be a statistical artifact. Secondly, competition is usually highest between dominant and subdominant species, but lower between dominant and subordinate species (Arnan et al., 2011). Hence, rare species, which are typically subordinate, may experience less pressure to partition their niches among each other. Andersen (2008) proposed that ant communities are to a significant extent a “lottery” system where colony establishment strongly depends on chance. Once a colony is established, it is very persistent and competition will not lead to nest mortality but will rather reduce performance (Andersen, 2008; Gordon & Kulig, 1996; Gordon & Wagner, 1997). Furthermore, rare species exert less pressure on each other since they occur at lower densities. In order to coexist, a rare species primarily has to differ from the dominant species, not from other rare ones. At high levels of competitive exclusion, a rare species’ chance to establish may be highly random, which further reduces the role of co‐occurring competitors and the need for niche partitioning. This idea is consistent with the highly competitive exclusion in PPF, which coincides with the lowest level of dietary and temporal differentiation (measures as percent explained variance).

## 5 CONCLUSION

All our rainforest ant communities showed substantial niche differentiation despite high niche overlap. In particular, each community contained species that foraged on less attractive food resources, indicating that relatively unattractive and low‐quality resources can be important for competitively inferior species, but also for niche partitioning among dominant ones. However, the relative importance of dietary and temporal niche differentiation varied markedly among our sites, despite their similar climate and vegetation structure. A mechanistic understanding of the global drivers of niche structure in ant communities therefore remains elusive. However, site‐specific idiosyncrasies appear to depend on traits of the locally dominant species, and so a fruitful avenue for future studies is to determine how ecological traits of dominant species affect niche structure and spatial segregation, and to understand the causes of trait variation in dominant species.

## CONFLICT OF INTEREST

None declared.

## AUTHOR CONTRIBUTIONS

FM conceived the study. MH and MG performed the fieldwork. MG and FM analyzed the data. AA supervised the Australian fieldwork and provided his expertise. MG and FM wrote the manuscript. All authors helped to improve the manuscript.

## DATA ACCESSIBILITY

Data of species occurrences per food resource, time of day, and grid point for each site are available online from the Dryad Digital Repository: https://doi.org/10.5061/dryad.1hj8q5q.

## References

[ece35394-bib-0001] Agosti, D. , & Alonso, L. . (2000) The ALL protocol In Agosti D., Majer J., Alonso E. & Schultz T. R. (Eds.), Ants: Standard methods for measuring and monitoring biodiversity (pp. 204–206). Washington, DC: Smithsonian Institution Press.

[ece35394-bib-0002] Albrecht, M. , & Gotelli, N. J. (2001). Spatial and temporal niche partitioning in grassland ants. Oecologia, 126, 134–141. 10.1007/s004420000494 28547432

[ece35394-bib-0003] Andersen, A. N. (2008). Not enough niches: Non‐equilibrial processes promoting species coexistence in diverse ant communities. Austral Ecology, 33, 211–220. 10.1111/j.1442-9993.2007.01810.x

[ece35394-bib-0004] Andersen, A. N. , Arnan, X. , & Sparks, K. (2013). Limited niche differentiation within remarkable co‐occurrences of congeneric species: *Monomorium* ants in the Australian seasonal tropics. Austral Ecology, 38, 557–567. 10.1111/aec.12000

[ece35394-bib-0005] Anjos, D. V. , Caserio, B. , Rezende, F. T. , Ribeiro, S. P. , Del‐Claro, K. , & Fagundes, R. (2017). Extrafloral‐nectaries and interspecific aggressiveness regulate day/night turnover of ant species foraging for nectar on *Bionia coriacea* . Austral Ecology, 42, 317–328. 10.1111/aec.12446

[ece35394-bib-0006] Arnan, X. , Gaucherel, C. , & Andersen, A. N. (2011). Dominance and species co‐occurrence in highly diverse ant communities: A test of the interstitial hypothesis and discovery of a three‐tiered competition cascade. Oecologia, 166, 783–794. 10.1007/s00442-011-1919-y 21290149

[ece35394-bib-0007] Baccaro, F. B. , De Souza, J. L. P. , Franklin, E. , Lemes landeiro, V. , & Magnusson, W. E. (2012). Limited effects of dominant ants on assemblage species richness in three Amazon forests. Ecological Entomology, 37, 1–12. 10.1111/j.1365-2311.2011.01326.x

[ece35394-bib-0008] Bernstein, R. A. (1979). Schedules of foraging activity in species of ants. Journal of Animal Ecology, 48, 921–930. 10.2307/4204

[ece35394-bib-0009] Blaimer, B. B. , Brady, S. G. , Schultz, T. R. , & Fisher, B. L. . (2015). Functional and phylogenetic approaches reveal the evolution of diversity in a hyper diverse biota. Ecography, 38(9), 901–912. 10.1111/ecog.01370.

[ece35394-bib-0010] Blüthgen, N. , & Feldhaar, H. (2010). Food and shelter: How resources influence ant ecology In Lach L., Parr C. L. & Abbott K. L. (Eds.), Ant Ecology (pp. 115–136). New York, NY: Oxford University Press.

[ece35394-bib-0011] Blüthgen, N. , & Fiedler, K. (2004a). Competition for composition: Lessons from nectar‐feeding ant communities. Ecology, 85, 1479–1485. 10.1890/03-0430

[ece35394-bib-0012] Blüthgen, N. , & Fiedler, K. (2004b). Preferences for sugars and amino acids and their conditionality in a diverse nectar‐feeding ant community. Journal of Animal Ecology, 73, 155–166. 10.1111/j.1365-2656.2004.00789.x

[ece35394-bib-0013] Blüthgen, N. , Gebauer, G. , & Fiedler, K. (2003). Disentangling a rainforest food web using stable isotopes: Dietary diversity in a species‐rich ant community. Oecologia, 137, 426–435. 10.1007/s00442-003-1347-8COMMUNITY 12898386

[ece35394-bib-0014] Bolnick, D. I. (2001). Intraspecific competition favours niche width expansion in *Drosophila melanogaster* . Nature, 410, 463–466. 10.1038/35068555 11260712

[ece35394-bib-0015] Bolnick, D. I. , Ingram, T. , Stutz, W. E. , Snowberg, L. K. , Lau, O. L. , & Paull, J. S. (2010). Ecological release from interspecific competition leads to decoupled changes in population and individual niche width. Proceedings of the Royal Society B‐Biological Sciences, 277, 1789–1797. 10.1098/rspb.2010.0018 PMC2871882 20164100

[ece35394-bib-0016] Brühl, C. A. , Gunsalam, G. , & Linsenmair, K. E. (1998). Stratification of ants (Hymenoptera, Formicidae) in a primary rain forest in Sabah, Borneo. Journal of Tropical Ecology, 14, 285–297. 10.1017/S0266467498000224

[ece35394-bib-0017] Carroll, C. , & Janzen, D. (1973). Ecology of foraging by ants. Annual Review of Ecology and Systematics, 4, 231–257. 10.1146/annurev.es.04.110173.001311

[ece35394-bib-0018] Chase, J. M. , & Leibold, M. A. (2003). Ecological niches: Linking classical and contemporary approaches. Chicago, IL: University of Chicago Press.

[ece35394-bib-0019] Chew, R. M. (1977). Some ecological characteristics of the ants of a desert‐shrub community in Southeastern Arizona. American Midland Naturalist, 98, 33–49. 10.2307/2424713

[ece35394-bib-0020] Davidson, D. W. (1977). Species diversity and community organization in desert seed‐eating ants. Ecology, 58, 711–724. 10.2307/1936208

[ece35394-bib-0021] Davidson, D. W. , Cook, S. C. , & Snelling, R. R. (2004). Liquid‐feeding performances of ants (Formicidae): Ecological and evolutionary implications. Oecologia, 139, 255–266. 10.1007/s00442-004-1508-4 15034777

[ece35394-bib-0022] Development Core Team, R. (2016). R: A language and environment for statistical computing. Vienna Austria: R Found Stat Comput 0:{ISBN} 3‐900051‐07‐0. 10.1038/sj.hdy.6800737

[ece35394-bib-0023] Devoto, M. , Bailey, S. , & Memmott, J. (2011). The “night shift”: Nocturnal pollen‐transport networks in a boreal pine forest. Ecological Entomology, 36, 25–35. 10.1111/j.1365-2311.2010.01247.x

[ece35394-bib-0024] Donoso, D. A. (2014). Assembly mechanisms shaping tropical litter ant communities. Ecography (Cop), 37, 490–499. 10.1111/j.1600-0587.2013.00253.x

[ece35394-bib-0025] Ellwood, M. D. F. , Blüthgen, N. , Fayle, T. M. , Foster, W. A. , & Menzel, F. (2016). Competition can lead to unexpected patterns in tropical ant communities. Acta Oecologica, 75, 24–34. 10.1016/j.actao.2016.06.001

[ece35394-bib-0026] Feldhaar, H. , Gebauer, G. , & Blüthgen, N. (2010) Stable isotopes: past and future in exposing secrets of ant nutrition (Hymenoptera: Formicidae). Myrmecological News, 13, 3–13.

[ece35394-bib-0027] Fichaux, M. , Béchade, B. , Donald, J. , Weyna, A. , Delabie, J. H. C. , Murienne, J. , … Orivel, J. (2019). Habitats shape taxonomic and functional composition of Neotropical ant assemblages. Oecologia, 189, 501–513. 10.1007/s00442-019-04341-z 30701386

[ece35394-bib-0028] Floren, A. , & Linsenmair, K. E. (2005). The importance of primary tropical rain forest for species diversity: An investigation using arboreal ants as an example. Ecosystems, 8, 559–567. 10.1007/s10021-002-0272-8

[ece35394-bib-0029] Folgarait, P. J. (1998). Ant biodiversity and its relationship to ecosystem functioning: A review. Biodiversity and Conservation, 7, 1221–1244.

[ece35394-bib-0030] Fowler, D. , Lessard, J. P. , & Sanders, N. J. (2014). Niche filtering rather than partitioning shapes the structure of temperate forest ant communities. Journal of Animal Ecology, 83, 943–952. 10.1111/1365-2656.12188 24289457

[ece35394-bib-0031] Gordon, D. M. , & Kulig, A. W. (1996). Founding, foraging, and fighting: Colony size and the spatial distribution of harvester ant nests. Ecology, 77, 2393–2409. 10.2307/2265741

[ece35394-bib-0032] Gordon, D. M. , & Wagner, D. (1997). Neighborhood density and reproductive potential in harvester ants. Oecologia, 109, 556–560. 10.1007/s004420050116 28307339

[ece35394-bib-0033] Gotelli, N. J. , & Ellison, A. M. (2002). Biogeography at a regional scale : Determinants of ant species density in New England bogs and forests. Ecology, 83, 1604–1609. 10.1890/0012-9658(2002)083[1604:BAARSD]2.0.CO;2

[ece35394-bib-0034] Gotelli, N. J. , & Entsminger, G. L. (2004). EcoSim: Null models software for ecology. Version 7. Jericho, VT: Acquired Intelligence Inc. and Kesey‐Bear.

[ece35394-bib-0035] Harvey, E. S. , Dorman, S. R. , Fitzpatrick, C. , Newman, S. J. , & McLean, D. L. (2012). Response of diurnal and nocturnal coral reef fish to protection from fishing: An assessment using baited remote underwater video. Coral Reefs, 31, 939–950. 10.1007/s00338-012-0955-3

[ece35394-bib-0036] Hölldobler, B. (1983). Territorial behavior in the green tree ant (*Oecophylla smaragdina*). Biotropica, 15, 241 10.2307/2387648

[ece35394-bib-0037] Hölldobler, B. , & Wilson, E. O. (1990). The ants. Cambridge, MA: Harvard University Press.

[ece35394-bib-0038] Houadria, M. , Blüthgen, N. , Salas‐Lopez, A. , Schmitt, M.‐I. , Arndt, J. , Schneider, E. , … Menzel, F. (2016). The relation between circadian asynchrony, functional redundancy, and trophic performance in tropical ant communities. Ecology, 97, 225–235. 10.1890/14-2466.1.The 27008791

[ece35394-bib-0039] Houadria, M. , & Menzel, F. (2017). What determines the importance of a species for ecosystem processes? Insights from tropical ant assemblages. Oecologia, 184, 885–899. 10.1007/s00442-017-3900-x 28744571

[ece35394-bib-0040] Houadria, M. , Salas‐Lopez, A. , Orivel, J. , Blüthgen, N. , & Menzel, F. (2015). Dietary and temporal niche differentiation in tropical ants — Can they explain local ant coexistence? Biotropica, 47, 208–217. 10.1111/btp.12184

[ece35394-bib-0041] Hutchinson, G. E. (1959). Homage to santa rosalia or Why are there so many kinds of animals? American Naturalist, 93, 145–159.

[ece35394-bib-0042] Junker, R. R. , Höcherl, N. , & Blüthgen, N. (2010). Responses to olfactory signals reflect network structure of flower‐visitor interactions. Journal of Animal Ecology, 79, 818–823. 10.1111/j.1365-2656.2010.01698.x 20412348

[ece35394-bib-0043] Kaspari, M. , & Weiser, M. D. (2000). Ant activity along moisture gradients in a neotropical forest. Biotropica, 32, 703–711. 10.1646/0006-3606(2000)032

[ece35394-bib-0044] Kay, A. (2004). The relative availabilities of complementary resources affect the feeding preferences of ant colonies. Behavioral Ecology, 15, 63–70. 10.1093/beheco/arg106

[ece35394-bib-0045] Kingston, T. , Jones, G. , Zubaid, A. , & Kunz, T. H. (2000). Resource partitioning in rhinolophoid bats revisited. Oecologia, 124, 332–342. 10.1007/PL00008866 28308770

[ece35394-bib-0046] Knaden, M. , & Wehner, R. (2005). Coexistence of two large‐sized thermophilic desert ants: The question of niche differentiation in *Cataglyphis bicolor* and *Cataglyphis mauritanica*. (Hymenoptera). Myrmecological News, 7, 31–42.

[ece35394-bib-0047] Leibold, M. A. , & McPeek, M. A. (2006). Coexistence of the niche and neutral perspectives in community ecology. Ecological Society of America: Issues in Ecology, 87, 1399–1410. 10.1890/0012-9658(2006)87[1399:cotnan]2.0.co;2 16869414

[ece35394-bib-0048] Lovette, I. J. , & Hochachka, W. M. (2006). Simultaneous effects of phylogenetic niche conservatism and competition on avian community structure. Ecology, 87, 14–28. 10.1890/0012-9658(2006)87[14:SEOPNC]2.0.CO;2 16922299

[ece35394-bib-0049] Lynch, J. F. , Balinsky, E. C. , & Vail, S. G. (1980). Foraging patterns in three sympatric forest ant species, *Prenolepis imparis*, *Paratrechina melanderi* and *Aphaenogaster rudis* (Hymenoptera: Formicidae). Ecological Entomology, 5, 353–371. 10.1111/j.1365-2311.1980.tb01160.x

[ece35394-bib-0050] Macarthur, R. , & Levins, R. (1967). The Limiting similarity, convergence, and divergence of coexisting species. American Naturalist, 101, 377.

[ece35394-bib-0051] Maret, T. T. , & Collins, J. P. (1997). Ecological origin of morphological diversity: A study of alternative trophic phenotypes in larval salamanders. Evolution (N Y), 51, 898–905. 10.2307/2411164 28568596

[ece35394-bib-0052] McKane, R. B. , Johnson, L. C. , Shaver, G. R. , Nadelhoffer, K. J. , Rastetter, E. B. , Fry, B. , … Murray, G. (2002). Resource‐based niches provide a basis for plant species diversity and dominance in arctic tundra. Nature, 415, 68–71. 10.1038/415068a 11780117

[ece35394-bib-0053] Menzel, F. , Staab, M. , Chung, A. Y. C. , Gebauer, G. , & Blüthgen, N. (2012). Trophic ecology of parabiotic ants: Do the partners have similar food niches? Austral Ecology, 37, 537–546. 10.1111/j.1442-9993.2011.02290.x

[ece35394-bib-0054] Mezger, D. , & Pfeiffer, M. (2011). Partitioning the impact of abiotic factors and spatial patterns on species richness and community structure of ground ant assemblages in four Bornean rainforests. Ecography (Cop), 34, 39–48. 10.1111/j.1600-0587.2010.06538.x

[ece35394-bib-0055] Mill, A. E. (1984). Predation by the ponerine ant *Pachycondyla commutata* on termites of the genus *Syntermes* in Amazonian rain forest. Journal of Natural History, 18, 405–410. 10.1080/00222938400770341

[ece35394-bib-0056] Nation, J. L. (2002). Insect physiology and biochemistry. Boca Raton, FL: CRC Press.

[ece35394-bib-0057] Ness, J. , Mooney, K. , & Lach, L. (2010). Ants as mutualits In Lach L., Parr C. & Abbott K.(Eds.), Ant ecology (pp. 97–114). Oxford, UK: Oxford University Press .

[ece35394-bib-0058] Parr, C. L. , & Gibb, H. (2010). Competition and the role of dominant ants. Oxford, UK: Oxford University Press.

[ece35394-bib-0059] Parr, C. L. , & Gibb, H. (2012). The discovery‐dominance trade‐off is the exception, rather than the rule. Journal of Animal Ecology, 81, 233–241. 10.1111/j.1365-2656.2011.01899.x 21854375

[ece35394-bib-0060] Philpott, S. , & Armbrecht, I. (2006). Biodiversity in tropical agroforests and the ecological role of ants and ant diversity in predatory function. Ecological Entomology, 31, 369–377. 10.1111/j.1365-2311.2006.00793.x

[ece35394-bib-0061] Philpott, S. M. , Perfecto, I. , Armbrecht, I. , & Parr, C. L. (2010). Ant diversity and function in disturbed and changing habitats In Lach L., Parr C. & Abbott K.(Eds.), Ant ecology (pp. 137–156). Oxford, UK: Oxford University Press .

[ece35394-bib-0062] Pianka, E. R. (1973). The structure of lizard communities. Annual Review of Ecology and Systematics, 4, 53–74. 10.1146/annurev.es.04.110173.000413

[ece35394-bib-0063] Quinlan, R. J. , & Cherrett, J. M. (1979). The role of fungus in the diet of the leaf‐cutting ant *Atta cephalotes* (L.). Ecological Entomology, 4, 151–160. 10.1111/j.1365-2311.1979.tb00570.x

[ece35394-bib-0064] Sanders, N. J. , Lessard, J. P. , Fitzpatrick, M. C. , & Dunn, R. R. (2007). Temperature, but not productivity or geometry, predicts elevational diversity gradients in ants across spatial grains. Global Ecology and Biogeography, 16, 640–649. 10.1111/j.1466-8238.2007.00316.x

[ece35394-bib-0065] Santamaria, C. , Armbrecht, I. , & Lachaud, J. (2009). Nest distribution and food preferences of Ectatomma ruidum (Hymenoptera: Formicidae) in shaded and open cattle pastures of Colombia. Sociobiology, 53, 517–542.

[ece35394-bib-0066] Santini, G. , Tucci, L. , Ottenetti, L. , & Frizzi, F. (2007). Competition trade‐offs in the organisation of a Mediterranean ant assemblage. Ecological Entomology, 32, 319–326. 10.1111/j.1365-2311.2007.00882.x

[ece35394-bib-0067] Stuble, K. L. , Rodriguez‐Cabal, M. A. , McCormick, G. L. , Jurić, I. , Dunn, R. R. , & Sanders, N. J. (2013). Tradeoffs, competition, and coexistence in eastern deciduous forest ant communities. Oecologia, 171, 981–992. 10.1007/s00442-012-2459-9 23242423

[ece35394-bib-0068] Tanaka, H. O. , Yamane, S. , & Itioka, T. (2010). Within‐tree distribution of nest sites and foraging areas of ants on canopy trees in a tropical rainforest in Borneo. Population Ecology, 52, 147–157. 10.1007/s10144-009-0172-2

[ece35394-bib-0069] Torres, J. A. (1984). Niches and coexistence of ant communities in Puerto Rico: Repeated patterns. Biotropica, 16(4), 284–295. 10.2307/2387937

[ece35394-bib-0070] Völkl, W. , Woodring, J. , Fischer, M. , Lorenz, M. W. , & Hoffmann, K. H. (1999). Ant‐aphid mutualisms: The impact of honeydew production and honeydew sugar composition on ant preferences. Oecologia, 118, 483–491. 10.1007/s004420050751 28307416

