# Supplementary material for: Niche differentiation in rainforest ant communities across three continents
Source: Ecol Evol. 2019 Jul 17;9(15):8601–15. doi: 10.1002/ece3.5394 (PMC6686352; doi:10.1002/ece3.5394)
Supplement: Supplementary file 1 [file ECE3-9-8601-s001.pdf]

Supporting information

Niche differentiation in rainforest ant communities across three continents

Short title: Niche differentiation of ant communities

Grevé, M.E. [1,2*], Houadria, M. [1,3], Andersen, A.N.[4,5], Menzel, F[1]

Author affiliation:

[1]University of Mainz, Institute of Zoology, J.-v.-Müller-Weg 5, 55099 Mainz, Germany

[2]University of Bayreuth, Animal Population Ecology, Animal Ecology I, Bayreuth Center of Ecology and Environmental Research (BayCEER), Universitätsstrasse 30, 95440 Bayreuth, Germany

[3]Institute of Entomology, Biology Centre of Academy of Sciences and Faculty of Science, University of South Bohemia, Branisovska, Ceske Budjovice, Czech Republic

[4]Tropical Ecosystems Research Centre, CSIRO Ecosystem Sciences, AMF, Australia

[5]Research Institute for the Environment and Livelihoods, Charles AMF University, AMF, Australia

* corresponding author. E-mail: michael.greve@uni-bayreuth.de

Supplementary methods: description of study sites

In Australia, the AMF was the Holmes Jungle nature reserve a patch of monsoon vine forests, 15 km northeast from Darwin, surrounded by savanna woodland and sedgeland (size: ~ 5 ha; 12' 25 'S, 130'' 58 'E). Sampling was conducted between October and December 2014 by M. Grevé. The climate in Darwin is tropical with dry (Mai to September) and wet season (December to March). The mean annual rainfall is approximately 1700 mm, with 80% falling between December and March. In the wet season, average temperatures range from 24.7°C to 32°C with 80% humidity, in the dry season, average temperatures range from 21.6°C to 31.8°C with 60-65% humidity.

In Sabah, Malaysian Borneo, the PPF was the primary lowland forest in the Danum Valley Conservation Area (size: ~ 438 km²; 04° 53.800' N, 117° 41.791' E), around 70 km east from Lahad Datu (Sabah). Sampling was conducted between Mai and June 2013 by M. Houadria, M. Schmitt and J. Arndt. The PSF was the Malua forest reserve around the Malua Field Center (size: ~ 350 km²; 04° 57.084' N, 117° 55.964' E) between August and September 2013 by M. Houadria and E. Schneider. The climate in Sabah is relatively aseasonal, with mean annual precipitation of 2880 mm and 80-90% relative humidity. Daily temperatures range from 19 to 34°C (annual mean: 26.9°C). Rainfall tends to be highest from November to February.

In French Guyana, the NPF was the primary lowland forest was the area around the "Les Nouragues Inselberg"-station at the foot of the Inselberg, a part of the Guiana Shield at the Nouragues Natural reserve (size: > 1000 km²; 4°05'14.49" N, 52°40´48-73" W). Sampling was conducted during August to September 2012 by M. Houadria. The NSF was a secondary forest fragment (size: ~ 16 ha; 5°10´18.56" N 52°39´18.22" W) within Kourou, surrounded by urban grass and residential areas. Sampling was conducted from February to March 2012 by M. Houadria and A. Salas-Lopez. The climate in French Guyana is equatorial, with mean precipitation of 3000 mm and 80-90% relative humidity. Daily temperatures range from 20 to 33°C (annual mean: 27°C). The wet season is from December to August (with May as wettest month), often interrupted by a short, drier period (approximately a month) around March.

In all sites, sampling was conducted only in the absence of rain.

Table S1 Food preferences and link temperature of species (incidence >= 5) of each site in declining order. Species, which together account for 80% of all frequencies on food sources are highlighted in grey). The food sources are sorted by its attractiveness in declining order (see Fig 4a). Based on null model randomizations a food source was defined as absolutely preferred (green) if a species foraged on it significantly more often than other on other food sources, and as relatively preferred (red) if a species foraged significantly more often on it than the other species within its community.

**AMF**	Sucrose	Melezitose	crushed insects	Small prey	Seeds	Large prey	Bird feces
Pheidole sp.A	green		green	red	red		
Nylanderia sp.A	green, red	green, red	green				
Oecophylla smaragdina			green			red	
Odontomachus sp.A							
Camponotus sp.A							red
Camponotus sp.B							
Pheidole sp.B							
Carebara sp.A					green, red		
Tetramorium sp.A							
Calomyrmex sp.A							
Monomorium sp.A							

**PPF**	Sucrose	Melezitose	crushed insects	Small prey	Seeds	Large prey	Bird feces
Lophomyrmex bedoti	green	green	green, red				
Carebara sp.1	green				red		
Lophomyrmex longicornis							
Nylanderia sp.4	green	green, red					
Tapinoma sp.1	green						
Pheidole sp.6		green			red		
Pheidole sp.5							
Euprenolepis sp.1							
Recurvidris sp.2							
Pheidole sp.40							
Dinomyrmex gigas							red
Carabera sp.8							
Recurvidris sp.1							
Crematogaster sp.1							
Pheidole sp.1							
Carabera sp.2	green						
Tetramorium sp.8							
Pheidole sp.2							
Carabera sp.3							
Pheidole sp.4							
Pheidole sp.9							
Technomyrmex sp.2							
Aphaenogaster sp.2							
Monomorium sp.2							
Pheidole sp.3							
Recurvidris sp.5		red					
Aphaenogaster sp.3							
Carabera sp.4							
Diacamma sp.1							

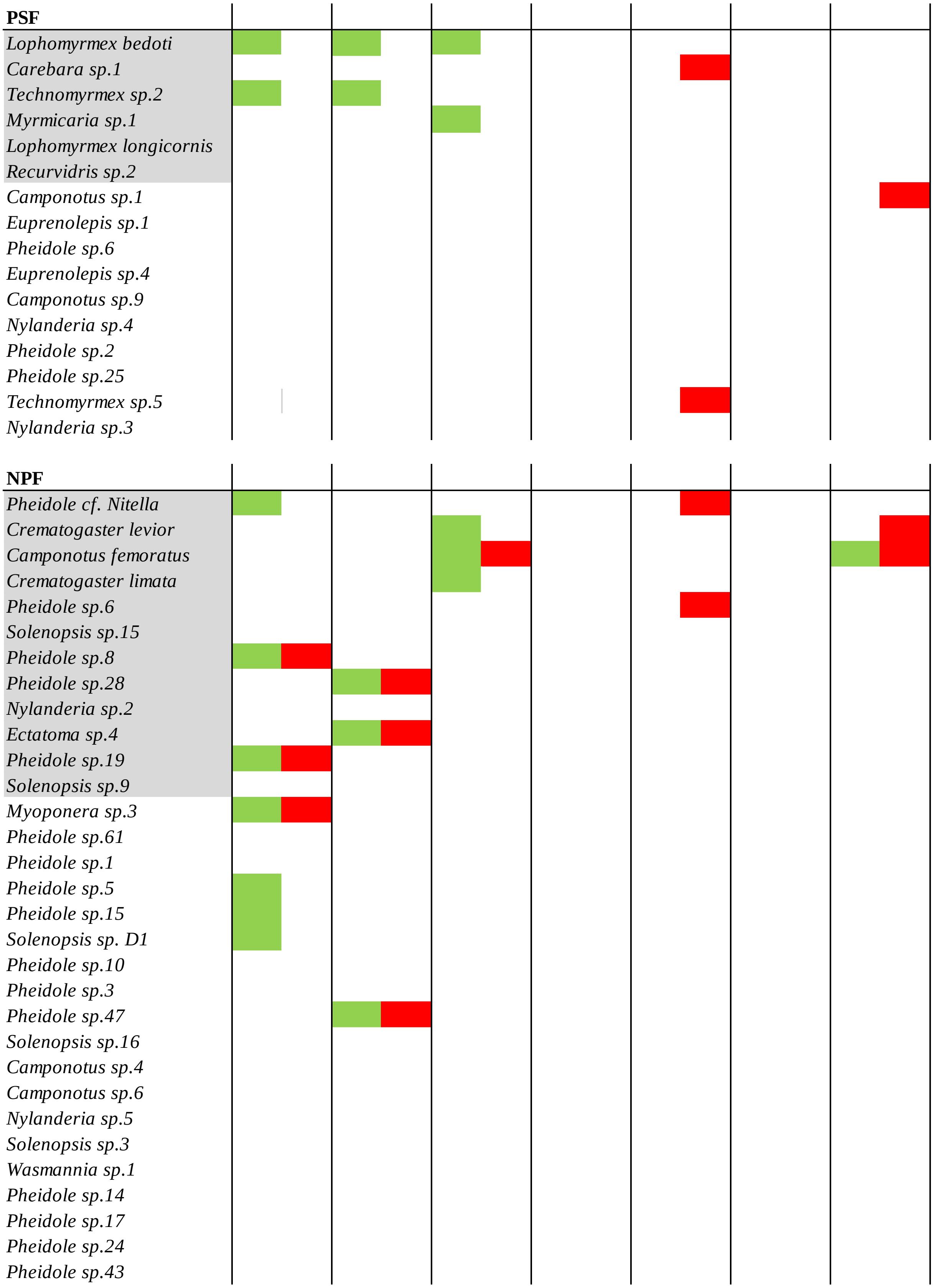
PSF
Lophomyrmex bedoti
Carebara sp.1
Technomyrmex sp.2
Myrmicaria sp.1
Lophomyrmex longicornis
Recurvidris sp.2
Camponotus sp.1
Euprenolepis sp.1
Pheidole sp.6
Euprenolepis sp.4
Camponotus sp.9
Nylanderia sp.4
Pheidole sp.2
Pheidole sp.25
Technomyrmex sp.5
Nylanderia sp.3
NPF
Pheidole cf. Nitella
Crematogaster levior
Camponotus femoratus
Crematogaster limata
Pheidole sp.6
Solenopsis sp.15
Pheidole sp.8
Pheidole sp.28
Nylanderia sp.2
Ectatoma sp.4
Pheidole sp.19
Solenopsis sp.9
Myoponera sp.3
Pheidole sp.61
Pheidole sp.1
Pheidole sp.5
Pheidole sp.15
Solenopsis sp. D1
Pheidole sp.10
Pheidole sp.3
Pheidole sp.47
Solenopsis sp.16
Camponotus sp.4
Camponotus sp.6
Nylanderia sp.5
Solenopsis sp.3
Wasmannia sp.1
Pheidole sp.14
Pheidole sp.17
Pheidole sp.24
Pheidole sp.43

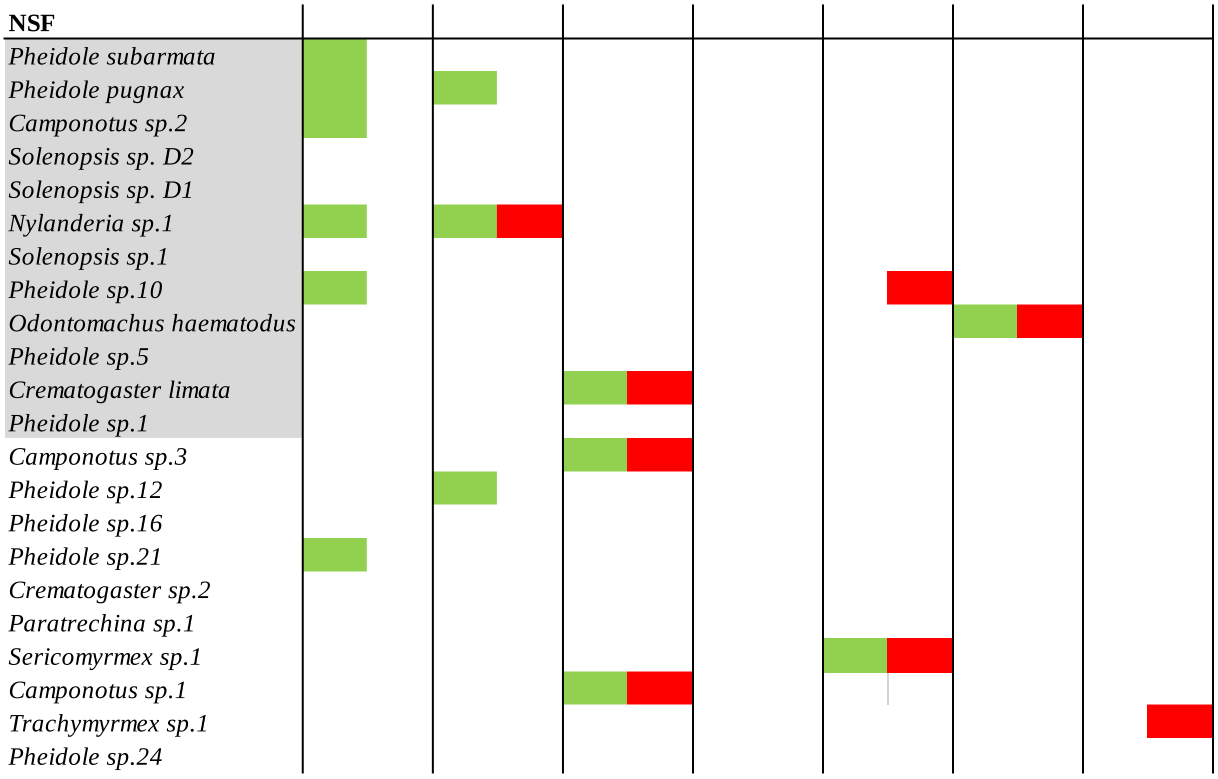
NSF
Pheidole subarmata
Pheidole pugnax
Camponotus sp.2
Solenopsis sp. D2
Solenopsis sp. D1
Nylanderia sp.1
Solenopsis sp.1
Pheidole sp.10
Odontomachus haematodus
Pheidole sp.5
Crematogaster limata
Pheidole sp.1
Camponotus sp.3
Pheidole sp.12
Pheidole sp.16
Pheidole sp.21
Crematogaster sp.2
Paratrechina sp.1
Sericomyrmex sp.1
Camponotus sp.1
Trachymyrmex sp.1
Pheidole sp.24

Table S2. Results of the (a) niche overlap analyses for the dietary and temporal niche and (b) co-occurrence analyses. The table shows the standardized effect size (SES), the observed index, the mean of the simulated indices and the p values for the observed index being smaller or larger than the simulated ones. Simulations were calculated using 1000 randomizations (niche overlap) and 5000 randomizations (C-score).

(a)

Site		SES	Observed mean niche overlap	Mean of simulated niche overlap	$p_{obs} < p_{sim}$	$p_{obs} > p_{sim}$
AMF	diet	4.662	0.531	0.445	1.00	**0.00**
	time	-0.756	0.763	0.777	0.03	0.97
PPF	diet	4.545	0.406	0.379	1.00	**0.00**
	time	1.792	0.742	0.729	0.94	0.06
PSF	diet	2.426	0.320	0.304	0.98	**0.02**
	time	-0.364	0.657	0.660	0.52	0.48
NPF	diet	10.881	0.465	0.393	1.00	**0.00**
	time	3.296	0.790	0.771	0.99	**0.01**
NSF	diet	5.398	0.555	0.506	1.00	**0.00**
	time	1.160	0.787	0.775	0.89	0.11

(b)

Site		SES	Observed C-score	Mean of simulated C-score	$p_{obs} < p_{sim}$	$p_{obs} > p_{sim}$
AMF	cooc	0.66	11.42	11.04	0.74	0.26
PPF	cooc	-0.57	14.15	14.26	0.27	0.73
PSF	cooc	-1.01	3.15	3.23	0.16	0.84
NPF	cooc	-2.11	9.05	9.28	**0.02**	0.98
NSF	cooc	-0.63	26.19	26.48	0.26	0.74

Table S3. Number and percentage of species with significant food preferences on each site. For each species n (all sites N = 109) with an incidence on baits ≥ 5, we calculated the food preferences by using day and night pooled frequency data per bait and species. For each species occurrences were permutated randomly 1000 times among the seven different baits. Then we compared this null model to the real frequencies per bait and defined them as "higher" when the real frequencies were higher ($p = 0.025$) on a given bait than the 95% confidence interval of the null model. If a species occurred more often on a bait than expected by random, it was defined as a species with significant absolute food preferences. Note that species numbers are higher than those for the hot link analysis (Table 2), since we included all species with incidence ≥ 5 compared to the set of species accounting for 80% of the occurrences used in Table 2.

**Site**	**AMF**	**PPF**	**PSF**	**NPF**	**NSF**
significant food preferences	4	6	3	13	12
no preferences	9	23	13	18	10
% of species with significant food preferences	36 %	21 %	19 %	42 %	55 %

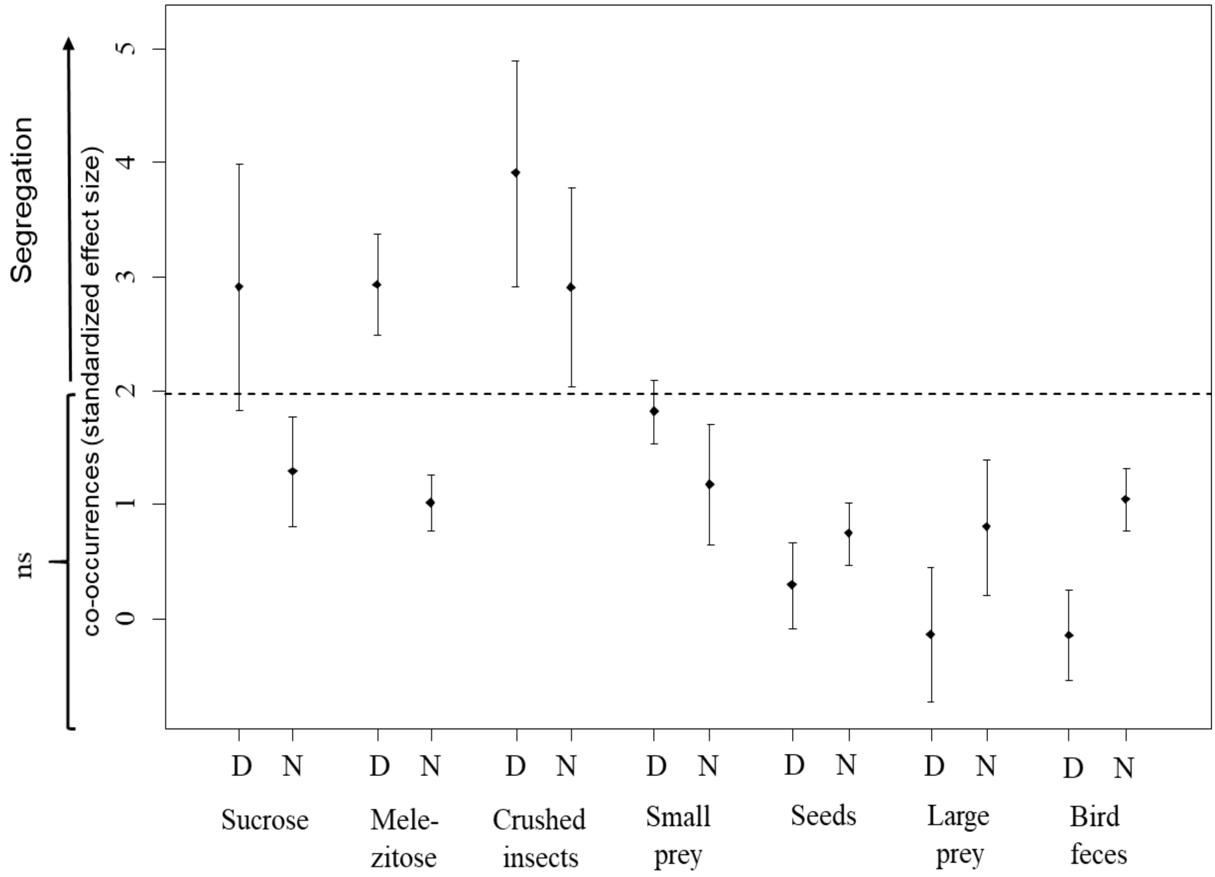

Fig. S1. Species co-occurrence on food resources separated for day (D) and night (N), shown as standardized effect sizes. Co-occurrence was calculated separately for each food source and time of day (n = 5 per food source/time of day). Values greater than 1.96 (dashed line) indicate significant species segregation.
